# mTOR signaling in Brown and Beige adipocytes: implications for thermogenesis and obesity

**DOI:** 10.1186/s12986-019-0404-1

**Published:** 2019-11-06

**Authors:** Yuqing Ye, Hailan Liu, Feng Zhang, Fang Hu

**Affiliations:** Department of Metabolism and Endocrinology, National Clinical Research Center for Metaboilc Diseases, Metabolic Syndrome Research Center, the Second Xiangya Hospital of Central South University, Changsha, Hunan China

**Keywords:** mTOR, Adipocytes, Thermogenesis, Energy metabolism

## Abstract

Brown and beige adipocytes are mainly responsible for nonshivering thermogenesis or heat production, despite the fact that they have distinguished features in distribution, developmental origin, and functional activation. As a nutrient sensor and critical regulator of energy metabolism, mechanistic target of rapamycin (mTOR) also plays an important role in the development and functional maintenance of adipocytes. While the recent studies support the notion that mTOR (mTORC1 and mTORC2) related signaling pathways are of great significance for thermogenesis and the development of brown and beige adipocytes, the exact roles of mTOR in heat production are controversial. The similarities and disparities in terms of thermogenesis might be ascribed to the use of different animal models and experimental systems, distinct features of brown and beige adipocytes, and the complexity of regulatory networks of mTORC1 and mTORC2 in energy metabolism.

## Introduction

There has been an accelerated global increase in the prevalence of obesity and obesity-related diseases, including metabolic syndrome and diabetes [1]. Recent data show that there are more than 1.9 billion overweight and over 650 million obese adults around the world in 2016 [2, 3]. Controlling body weight greatly benefits obesity associated chronic diseases and cancers [4–6]. Obesity mainly arises from an imbalance of energy homeostasis between increased food intake and decreased energy expenditure. The multiple functions of adipose tissues in energy homeostasis have been attracting increasing attention in recent years. Apart from their endocrine function, adipose tissues also play important roles in energy metabolism. As classical white adipose tissues (WATs) are mainly responsible for fat storage, brown adipose tissues (BATs) control nonshivering thermogenesis or heat production. BAT expresses unique uncoupling protein 1 (Ucp1), which allows the uncoupling protons to move down their mitochondrial gradient from the synthesis of adenosine triphosphate (ATP), resulting in the dissipation of energy as heat [7]. Recent studies have uncovered that, when induced by a cold environment or β-adrenergic receptor (βAR) activators, a subset of “brown-like” or “beige” cells in WAT are produced.

Similar to brown adipocytes, beige adipocytes are also involved in nonshivering thermogenesis and the dissipation of heat energy. Recently, extensive studies that explore the molecular mechanisms underlying the functional and developmental regulation of brown and beige fat have been performed [8]. Multiple transcriptional factors and cofactors, such as PRD1-BF1-RIZ1 homologous domain-containing 16 (PRDM16), peroxisome proliferator-activated receptor γ (PPARγ), PPARγ-coactivators-1α (PGC-1α), and many others, play key roles in the differentiation and development of brown and beige adipocytes [9, 10].

mTOR, known as mammalian or mechanistic target of rapamycin, is a Ser/Thr protein kinase that integrates internal and external signaling to regulate protein/lipid syntheses, cellular proliferation and metabolism, and autophagy. Recently, mTOR-related signaling pathways have been reported to play vital roles in the regulation of adipose tissue browning and thermogenesis; however, the exact role of mTOR signaling in the beigeing process seems controversial and is yet to be fully elucidated. In this review, we summarize and highlight the recent understanding of adipose tissue browning. More importantly, we discuss the discoveries concerning mTOR-related signaling in brown and beige fat development and its function in different mouse models. We believe that clarifying the roles and underlying mechanisms of mTOR-related signaling in adipocytes will provide potential therapeutic targets for obesity and related metabolic disorders.

## Brown and beige adipocytes

As the two major types of adipocytes that are responsible for nonshivering thermogenesis, brown and beige adipocytes share multiple similarities in morphological and biochemical characteristics, namely, they both possess multilocular lipid droplets, a large number of mitochondria, and high expression levels of several thermogenic genes and increased dissipation of energy under cold stimulation or via pathways that elevate intracellular cyclic AMP [8]. With respect to their noncanonical function, brown and beige fat influence systemic metabolism indirectly by acting as metabolic sinks for various substrates, such as glucose, lipids, and many other metabolites [11, 12]. Despite similarities, these two kinds of adipocytes still have several distinctive features.

First, there is a difference in the distribution of brown and beige adipocytes. In human infants, classical brown adipocytes mainly exist in interscapular BAT depots, similar to those of rodents [13]. In human adults, BAT is more widely distributed. Its locations include the cervical, supraclavicular, axillary, paravertebral, and periadrenal regions [13, 14]. Intriguingly, adult human BAT depots express several molecular markers similar to beige adipocytes in mice, such as the homeobox protein HoxC8 (Hoxc8), the homeobox protein HoxC9 (Hoxc9) and the Cebp/p300-interacting transactivator with Glu/Asp-rich carboxy-terminal domain 1 (CITED1); thus, Ucp1-positive adipocytes from the supraclavicular region in humans show a molecular signature consistent with those of beige adipocytes in mice [15]. Unlike the dedicated deposits of brown adipocytes, inducible beige adipocytes are highly dependent on adipose depots. In mice, the subcutaneous inguinal WAT depots, such as the inguinal and anterior subcutaneous WATs, undergo profound induction [13, 16], whereas the epididymal WATs are particularly resistant to beigeing when exposed to cold stimuli [17].

Second, the developmental origin of classical brown and beige adipocytes is different. During the embryogenesis of mice, BAT depots develop before other adipose depots, which is in parallel with the capacity for nonshivering thermogenesis in a cold environment in newborns [18]. Lineage-tracing studies indicate that brown adipose precursors arise from multipotent progenitor cells in the dermomyotome that express high levels of engrailed 1 (EN1), paired box 7 (PAX7), and myogenic factor 5 (MYF5) marker genes, and such multipotent progenitors can also differentiate into skeletal muscle, dorsal dermis, and a subset of white adipocytes [19–21]. The committed brown adipose precursors develop into brown preadipocytes that express early B-cell factor 2 (EBF2; also known as COE2) [22]. Under the regulation of PRDM16, which interacts with adipogenic transcription factors CCAAT/enhancer-binding protein-β (C/EBPβ), PPARγ, zinc finger protein 516 (ZFP516), and euchromatic histone-lysine N-methyltransferase 1 (EHMT1), the brown preadipocytes eventually transform into classical brown adipocytes characterized by high levels of Ucp1, PGC-1α, and PRDM16 [8, 19, 23–25].

However, the origin of beige adipocytes in subcutaneous WAT is still disputed and as the sources of beige cells, distinct WAT depots display multivariable developmental origins. Beige adipocytes in inguinal subcutaneous WAT are reported to be derived from smooth muscle cells expressing smooth muscle actin (SMA), myosin heavy chain 11 (MYH11), and mural cells expressing platelet-derived growth factor receptor-β (PDGFRβ) [25]. SMA+ cells commit to preadipocytes with platelet-derived growth factor receptor-α (PDGFRα) and EBF2, and when under the modulation of PRDM16, PDGFRα, PPARγ, or ZFP516, preadipocytes differentiate into beige adipocytes [26–30]. However, another possible origin of beige adipocytes in inguinal subcutaneous WAT is the interconversion of mature white adipocytes under cold or β-adrenergic signaling stimulation, in which beige adipocytes can turn back into white adipocytes after warm adaptation [31]. In epididymal WAT, bipotent PDGFRα+ precursors can be converted into white adipocytes under a high-fat diet, whereas cold exposure or β-adrenergic stimulation leads to the differentiation of these precursor cells into beige adipocytes [32].

Third, unlike brown fat cells that express relatively higher levels of Ucp1 even under non-stimulated conditions, beige adipocytes are dependent on external stimuli for Ucp1 induction, which is a distinctive feature of beige cells [33]. In the basal state, beige adipocytes express a very low level of the thermogenic gene program that resembles white adipocytes; however, if fully stimulated, beige adipocytes express high levels of Ucp1 similar to those of brown adipocytes and undergo Ucp1-mediated uncoupled respiration. Thus, the beige cell’s capacity to switch between energy storage and energy dissipation depends on the type of stimulation that it receives, a capacity that classic brown adipocytes lack [16, 25].

Given the above similarities between brown and beige adipocytes and the distinctive features that they possess, more investigations are necessary to identify the mechanisms and regulations of these two adipocytes in thermogenesis and energy homeostasis.

## mTOR signaling

**Fig. 1:**
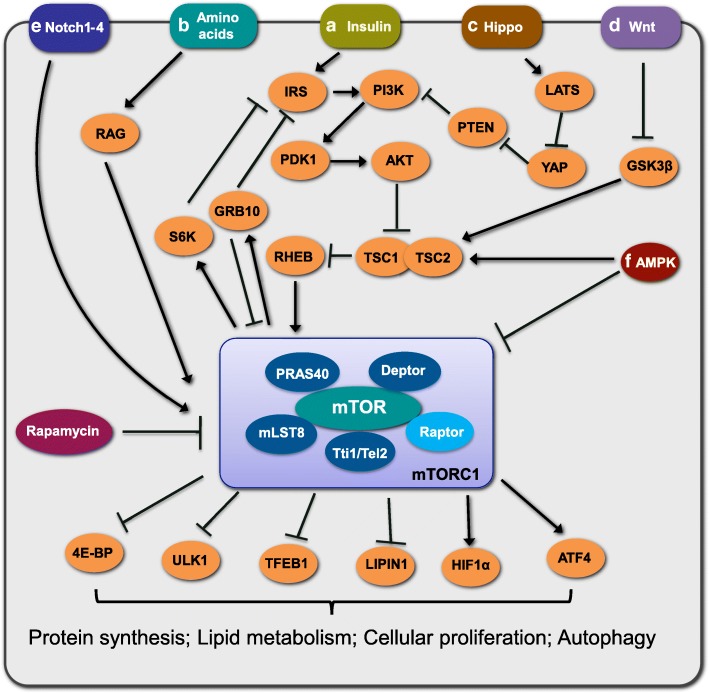
mTORC1 and its signaling networks. mTORC1 is composed of mTOR, Raptor, mLST8, PRAS40, Deptor and Tti1/Tel2 complex. The signaling networks of mTORC1. **a** Growth factors, such as insulin, stimulation leads to the activation of classic PI3K-AKT-TSC2-mTORC1 pathway. **b** Amino acids, mainly leucine and arginine, stimulate mTORC1 through GTP-loaded RAG. **c** LATS can trigger hippo pathway by inhibition of YAP to activate mTORC1 signaling through PTEN suppression. **d** WNT signaling stimulates mTORC1 by inhibiting the activation of GSK3β, which can phosphorylate TSC2. **e** Notch signaling can regulate mTOR activity in liver. **f** AMPK inhibits mTORC1 activity through phosphorylating TSC2 or Raptor under energy limitation. Upon various stimulation, mTORC1 modulates its substrates such as 4E-BP, ULK1, TFEB1, LIPIN1, HIF1α, ATF4. S6K and GRB10 to affect cellular proliferation, metabolism and many other biological processes. In addition, negative feedback mechanisms of mTOR substrates, in turn, fine-tune mTORC1 signaling networks. For examples, Grb10 negatively regulates the mTORC1 signaling pathway through a phosphorylation-dependent feedback mechanism on reptor or IRS. And S6K1 also negatively accommodates phosphorylation of IRS-1 to suppress IRS-1/PI3K /Akt /mTOR signaling. Akt, protein kinase B; AMPK, 5’AMP- activated protein kinase; ATF4, activating transcription factor 4; Deptor, DEP domain-containing mTOR-interacting protein; GRB10, growth factor receptor-bound protein 10; GSK3β, glycogen synthase kinase 3β; HIF1α, hypoxia inducible factor-1α; IRS, insulin receptor substrate; LATS, large tumour suppressor homologue kinase; mLST8, mammalian lethal with SEC13 protein 8; mTORC1, mammalian or mechanistic target of rapamycin complex1; PDK1, Phosphoinositide-dependent kinase-1; PI3K, phosphoinositide 3-kinase; PRAS40, Akt/PKB substrate 40 kDa; PTEN, phosphatase and tensin homolog; RAG, RAS-related GTP-binding Protein; Raptor, regulatory associated protein of mTOR; RHEB, RAS homolog enriched in brain; S6K, ribosomal S6 kinase; TFEB1, transcription factor EB; TSC1, tuberous sclerosis complex 1; TSC2, tuberous sclerosis complex 2; ULK1, UNC-like kinase 1; YAP, Yes-associated protein; 4E-BP, eIF4E-binding protein

**Fig. 2:**
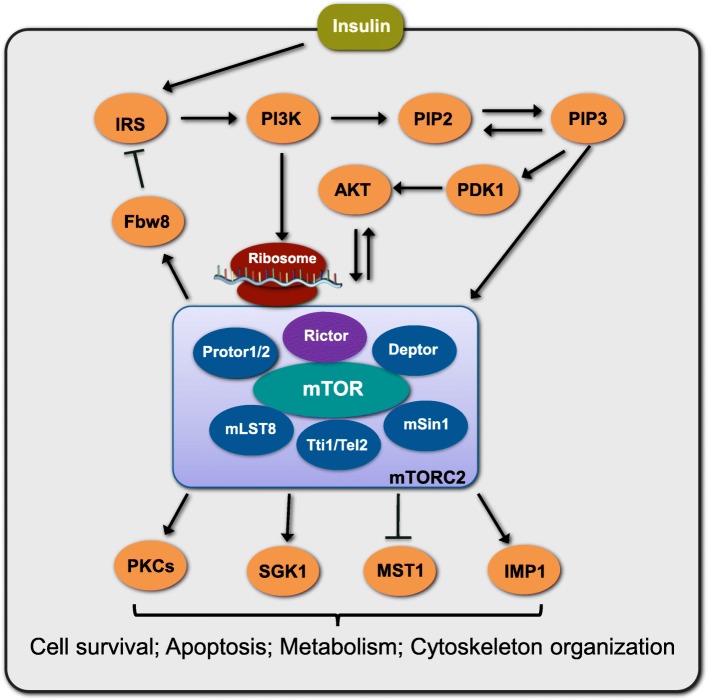
mTORC2 and its signaling networks mTORC2 is composed of mTOR, Deptor, Rictor, mLST8, mSin1, PROTOR1/2 and Tti1/Tel2 complex. The signaling networks of mTORC2. The classic growth factors such as insulin stimulation through PI3K signaling to promote mTORC2-ribosome binding and activation of mTORC2. Growth factor-dependent activation of PIP3 interacts with mSin1 to enhance the activity of mTORC2 and initiation of its downstream signaling. Akt, as a downstream of PDK1 can directly phosphorylate mSin1 thus activating mTORC2, which, in turn, positively feeds back to phosphorylate and activate Akt. Upon activation, mTORC2 phosphorylates its downstream substrates, including SGK1, PKC, MST1, IMP1 and Akt. mTORC2 can negatively feeds back to IRS through Fbw8. Deptor, DEP domain-containing mTOR-interacting protein; Fbw8, F-box/WD repeat-containing protein; IMP1, IGF2 mRNA-binding protein 1; IRS, insulin receptor substrate; mLST8, mammalian lethal with SEC13 protein 8; mSin1, mammalian stress-activated protein kinase-interacting protein 1; MST1, mammalian sterile 20-like kinase 1; mTORC2, mammalian or mechanistic target of rapamycin complex 2; PDK1, Phosphoinositide-dependent kinase-1;PIP2, phosphatidylinositol (4,5) bisphosphate; PIP3, phosphatidylinositol-(3,4,5)-trisphosphate; PI3K, phosphoinositide 3-kinase; PKC, protein kinase C; PROTOR1/2, protein observed with Rictor-1 and -2; Rictor, Raptor-independent companion of mTOR; SGK1, serum- and glucocorticoid-induced protein kinase 1

In order to modulate various biological processes, such as protein and lipid synthesis, cellular growth, proliferation, differentiation, and autophagy [34, 35], mTOR will respond to both intracellular and extracellular environmental changes. There are two biochemically and functionally distinct mTOR complexes, mTORC1 and mTORC2, both of which are composed of mTOR, which acts as the catalytic core of the complex. mTORC1 contains two core components, regulatory associated proteins of mTOR (Raptor) and mammalian lethal with SEC13 protein 8 (mLST8); two inhibitory subunits, Akt/PKB substrate 40 kDa (PRAS40) and DEP domain-containing mTOR-interacting protein (Deptor); and the stabilizing complex Tti1/Tel2 [36] (Fig. 1). mTORC2 shares mLST8, Deptor, and the Tti1/Tel2 complex with mTORC1 but has three unique elements: Raptor-independent companion of mTOR (Rictor), mammalian stress-activated protein kinase-interacting protein 1 (mSin1), and protein observed with Rictor-1 and -2 (PROTOR1/2) [36–38] (Fig. 2).

### mTORC1

mTORC1 is known as a critical regulator in metabolism, ribosomal biogenesis, cap-dependent translation, nucleotide biosynthesis, lysosomal biogenesis, lipid synthesis, autophagy, and thermogenesis. mTORC1 can be activated by many internal and external factors, including growth factors, amino acids, cellular energy status, stress, oxygen, and certain signaling pathways, such as WNT, Hippo, and Notch [39] (Fig. 1). Growth factors such as insulin-like growth factors and insulin bind to their respective receptors, thus activating PI3K/Akt signaling, triggering phosphorylation-mediated inhibition of tuberous sclerosis complex 1/2 (TSC 1/2), and leading to an increase in GTP-bound activation of Rheb, which directly activates mTORC1 [40, 41]. Akt can also phosphorylate PRAS40 to dissociate the latter from Raptor, thus resulting in the activation of mTORC1 [42]. Branched-chain amino acids such as leucine and arginine collaborate with RAS-related GTP-binding protein (Rag) and Raptor and promote the relocalization and activation of mTORC1 [43]. Large tumor suppressor homologue kinase (LATS) can trigger the hippo pathway by inhibiting Yes-associated protein (YAP), which then activates mTORC1 signaling through the suppression of phosphatase and tensin homologue (PTEN) [44]. WNT signaling stimulates mTORC1 by suppressing the activation of glycogen synthase kinase 3β (GSK3β), which can phosphorylate TSC2 [45]. Notch signaling can increase the stability of mTORC1 by promoting Akt activity and therefore increasing hepatic lipid accumulation [46]. Moreover, AMP-activated protein kinase (AMPK) inhibits mTORC1 activity through phosphorylating TSC2 or Raptor when cellular energy shortage occurs [47].

Upon activation, mTORC1 functions to phosphorylate its substrates and triggers a cascade of signaling transduction. Many direct substrates of mTORC1 have been identified, including ribosomal S6 kinase (S6K), eIF4E-binding proteins (4E-BPs), transcription factor EB (TFEB1), Lipin1, UNC-51-like kinase 1 (Ulk1), growth factor receptor-bound protein-10 (Grb10), hypoxia inducible factor-1α (HIF1α) and activating transcription factor 4 (ATF4) [48–53] (Fig. 1). Alternatively, negative feedback mechanisms of mTOR substrates could, in turn, fine-tune the mTOR signaling network. For example, Grb10 negatively regulates the mTORC1 signaling pathway through a phosphorylation-dependent feedback mechanism, and S6K1 also negatively accommodates phosphorylation of insulin receptor substrate 1 (IRS1) to suppress IRS-1/PI3K/Akt/mTOR signaling [54–57].

### mTORC2

Unlike mTORC1, mTORC2 is involved in cell survival, proliferation, apoptosis, metabolism, and cytoskeleton organization [58–60]. Although insensitive to acute rapamycin treatment, the activity of mTORC2 is inhibited by chronic treatment [58, 61]. Growth factor-stimulated PI3K signaling can promote mTORC2 activation and ribosome binding [62]. Similar to mTORC1, there is a negative feedback mechanism in mTORC2 signaling in coordination with its upstream molecules, such as IRS-1 [63]. In particular, Akt promotes the activation of mTORC2, which in turn positively feeds back to phosphorylate and activates Akt [64]. Strikingly, mTORC2 signaling is also regulated by mTORC1 through Grb10 in insulin/PI3K signaling [49, 55]. In addition, mTORC2 functions to phosphorylate several members of the AGC kinases, such as protein kinase C (PKC), serum- and glucocorticoid-induced protein kinase 1 (SGK1), IGF2 mRNA-binding protein 1 (IMP1), mammalian sterile 20-like kinase 1 (MST1), and Akt, to regulate cell survival, proliferation, and metabolism [58, 60, 65–70] (Fig. 2).

## mTOR-related signaling in brown and beige adipocytes

Accumulated evidence over the years has shown that mTOR is a key energy sensor and that its associated signaling pathways control lipid metabolism and adipocyte formation and maintenance [38]. Not only does the impairment of adipose mTOR signaling suppresses the development and expansion of the BATs and WATs [71–73], but it also influences the functions and metabolism of fat tissues. Recent studies have demonstrated that mTOR is also involved in the controlling of nonshivering thermogenesis and the development of brown/beige adipocytes. However, conflicting observations concerning the role of mTOR-related signaling pathways in thermogenic gene expression in adipocytes still exist. Also, the molecular mechanisms underlying mTOR-regulated thermogenesis and the development of brown/beige adipocytes are still poorly understood. In the next section, we summarize recent discoveries of mTOR-related signaling in the regulation of brown and beige adipocyte activation, and discuss the possibilities that cause discrepancies in the role of mTOR in thermogenesis.

### mTORC1 signaling in brown and beige adipocytes

mTORC1 is an important regulator of adipose tissue formation and lipogenesis. It has been suggested that mTORC1 plays an essential role in the regulation of adipocyte precursor commitment, adipogenesis (a process of preadipocyte differentiation into mature adipocytes), triacylglycerol (TAG) synthesis, and the mobilization in adipocytes [74]. Recently, an accumulating body of evidence has emerged which elucidates the effect of the mTORC1 signaling pathway on thermogenesis in brown and beige adipocytes.

#### Pharmacologic mTORC1 inhibition by rapamycin

Rapamycin is a well-recognized inhibitor of mTORC1. It acts by directly binding itself to the 12-kDa FK506- and rapamycin-binding protein (FKBP12, or FKBP) and the FKBP rapamycin-binding (FRB) domain of the mTOR kinase which disturbs the function of mTOR [75]. The effects of rapamycin on metabolism depend on the length of treatment: 2–6 weeks of rapamycin treatment produce detrimental metabolic changes that are usually associated with insulin resistance, hyperlipidemia and glucose intolerance [76, 77], whereas prolonged (20 weeks) rapamycin treatment causes better metabolic profiles with increased oxygen consumption and ketogenesis and enhanced insulin sensitivity [78].

As for the effects of the rapamycin treatment on thermogenesis and beige fat, the research of Liu et al. showed that, regardless of 2 days or 7 days of the rapamycin treatment, when responding to cold exposure, the expression of Ucp1 is diminished in both BAT and WAT [79] and in line with decreased core body temperature, although the rapamycin treatment alone has no such effects [79]. After observing mice that were treated with rapamycin for 2 weeks followed by a 24 h β3-adrenergic receptor (β3AR) agonist CL316,243 (CL) challenge [80], similar results were produced. However, for the unknown mechanism, CL treatment caused suppression of β3AR. The rapamycin treatment exacerbated this effect, which might have partially contributed to the suppressive effects of rapamycin on thermogenic gene expression [80]. These studies on mTORC1 inhibition by rapamycin seem to imply that mTORC1 plays a positive role in thermogenesis due to the fact that mTORC1 is capable of activating βAR signaling.

Interestingly, other observations show that diet-induced obese mice who underwent a chronic treatment (22 weeks) of rapamycin are not only leaner, but alsodisplay enhanced energy expenditure, oxygen consumption, and BAT activity, which might be associated with significant changes in the inflammatory profiles of adipose tissues — immune cells with regulatory functions, such as regulatory T cells (Tregs) and myeloid-derived suppressor cells, are increased in the adipose tissues [81]. This study is in accordance with previous research showing that mTOR inhibition could promote the generation of Tregs both in vitro and in vivo [82, 83]. The induction of Tregs under cold exposure, physiological levels of β-adrenergic stimulation, or high-caloric challenge is suggested to modulate thermogenesis and lipolysis advantageously both in BAT and beigeing of WAT through the Stat6/Pten axis, which is also involved in suppressed mTORC1 activity [84]. Thus, these studies seem to imply that the negative role that mTORC1 plays in the regulation of thermogenesis might be linked to its impact on Tregs in adipose tissues.

One plausible explanation for the different responses to rapamycin could be the duration of the treatment, either short-term/acute (2–7 days [79], 2 weeks [80]) or long-term/chronic (22 weeks [81]) treatment. Beneficial metabolic effects of rapamycin are consistently observed in long-term treatment [78, 81]. Although rapamycin inhibition of mTOR signaling is primarily due to its impact on mTORC1, prolonged rapamycin treatment may also affect mTORC2, whose impacts require further investigation. In addition, the effect of rapamycin treatment on lipid metabolism in vivo is still unclear, and it is difficult to explain the effects of whole-body rapamycin administration on lipolysis/lipogenesis and thermogenesis. Therefore, in order to address the function of mTORC1, tissue specific manipulation might be a more preferable approach.

#### Raptor deletion in adipose tissues

Studies on adipose tissue-specific deletion of one of the components of mTORC1 are vital in addressing the role of mTORC1 in thermogenesis and brown/beige fat development. Raptor is a 150-kDa mTOR binding protein that serves as a complex scaffold. The binding of Raptor to the motif of mTOR substrates is necessary for effective mTOR-catalyzed phosphorylation [85]. An earlier study using aP2-Cre to drive adipocyte-specific deletion of Raptor showed that Raptor^aP2-Cre^ mice have enhanced oxygen consumption and elevated basal levels of genes characteristic of brown fat, such as Ucp1, Dio2, and Cidea in WAT [86]. However, studies using adiponectin (Adipoq)-Cre showed that, when fed a normal diet, Raptor^Adipoq-Cre^ mice develop lipodystrophy associated with hepatomegaly, hepatic steatosis, and insulin intolerance. In addition, although mice display increased Ucp1 mRNA expression in WAT and are resistant to high-fat diet (HFD)-induced obesity, they do not have an increased energy expenditure [72]. Similar observations were also reported in mTOR adipose-deleted mice (mTOR^Adipoq-Cre^) [73]. In accordance with these discoveries, recently, the studies of Zhang et al. (2018) showed that the adipose-specific depletion of Raptor^Adipoq-Cre^ promotes beige adipogenesis, and mice are resistant to diet-induced obesity, possibly through prostaglandins (PGs) synthesized by cyclooxygenase-2 [87]. These studies [72, 73, 87] found that, although it suppressed the development and expansion of BAT and WAT, Raptor or mTOR fat-tissue-specific knockout induces basal Ucp1 expression and browning in WAT, possibly due to compensational effects of adipose loss. However, the aP2-Cre-generated mice showed no such phenotypes, and the underlying mechanism is unclear [86].

However, using the Adipoq-Cre model, Tran et al. (2016) demonstrated that fat Raptor^Adipoq-Cre^ mice show a decreased expression of Ucp1 under β3-adrenergic signaling stimulation but mild beigeing induction under unstimulated conditions [80]. Liu et al. reported that Raptor^Adipoq-Cre^ mice show impaired expression of Ucp1 and mitochondrial-related genes when exposed to cold or βAR agonists, but no change in Ucp1 expression at room temperature [79].

Although impaired adipose tissue development during postnatal growth was consistently observed, adipose Raptor ablation on thermogenesis and beige adipogenesis in vivo had opposing results, which might have multiple causes. One of these potential causes is the Cre model used in those studies. Polak et al. (2008) used an aP2-Cre-dependent model [86], whereas the remaining studies used Adipoq-Cre mice [71–73, 79, 80, 87]. The aP2-Cre model creates nonspecific deletions, as it is effective in both fat tissues and nonfat tissues, such as the brain, endothelium and other metabolic-related peripheral tissues (e.g., liver, skeleton muscle, etc.), and embryonic tissues [88–90]. Therefore, the influence from other Raptor-deleted tissues could not be ruled out. Because aP2 is also expressed in activated macrophages [91], as an infiltration of macrophages into adipose tissue, it has raised great concern, especially under overnutrition conditions, which may cause significant effects on local and systemic metabolism [92]. However, unlike aP2-Cre mice, non-adipose tissue recombination was not observed with Adipoq-Cre lines; therefore, Adiponectin-Cre is more specific and efficient at targeting mature adipocytes [90].

However, as both aP2 and adiponectin genes turn on at the early stage of development, observed phenotypes using these two Cre models might represent both developmental and physiological consequences of gene loss. For these reasons, it might be more appropriate to use an inducible (e.g., tamoxifen-induced) Cre system to avoid developmental stage-related effects and chronic effects of selective gene ablation. However, thus far, no inducible Cre system has been used to study the mTORC1-associated gene deletion in adipose tissues. In the future, to address the function of mTOR signaling in WAT in vivo, it is important that we use temporal control of the adipose-specific recombination system and the most updated and precise genetic and metabolic tools.

Another factor that could cause different results in those studies is the experimental system. The activity of beige adipose tissues is sensitive to the environmental temperature, and animals used in the above studies were generally housed at standard mouse facility (room) temperatures (often 22–23 °C) or severely cold temperatures (typically 4–10 °C), which induces “browning” of WAT. However, mice living at 22 °C are already cold stressed because this temperature is below their thermoneutral zone (30 °C), which could profoundly impact their basal metabolic rate [93]. In the above-mentioned adipose Raptor ablation studies, Raptor^Adipoq-Cre^ mice were either housed at room or thermoneutral temperatures or challenged by severe cold [79, 80, 87]. Increased Ucp1 expression in inguinal WAT (iWAT) was observed at room temperature and under thermoneutrality by Zhang et al. [87] and at room temperature by Lee et al. [72], but not by Liu et al. [79] and Tran et al. [80]. No differences in thermogenic gene expression in iWAT and O2 consumption were found between control and Raptor^Adipoq-Cre^ mice under cold stress conditions [87], whereas significantly reduced inductions of Ucp1 and other thermogenic genes were found in response to cold exposure [79] or β3AR agonist CL injection [80]. The different responses of Raptor^Adipoq-Cre^ mice to cold exposure might be caused by different experimental procedures with different durations of cold challenge. The mechanisms of short-term or long-term cold stress and β3-adrenergic signaling stimulation for thermogenesis are not completely identical, as cold might activate several redundant signaling pathways in addition to those linked to β3AR activation. For example, under cold stimulation, adipose-derived fibroblast growth factor 21 (FGF21) can act in an autocrine/paracrine manner to increase the expression of Ucp1 and other thermogenic genes in fat tissues, which is independent of the β3-adrenergic signaling pathway [94, 95].

Interestingly, unlike those discrepant observations in WAT, the adipose Raptor deletion causes consistent results in BAT that are different from those in WAT. The BAT mass and expression of Ucp1 were decreased in mice that were kept at room temperature [79, 87], and a similar blunted response of Ucp1 to severe cold was observed in BAT of Raptor^Adipoq-Cre^ [71, 79, 87], which might be due to the requirement of mTORC1 for BAT formation and maintenance that is linked to reduced nucleotide synthesis, mitochondrial biogenesis, and impaired TCA cycle activity [71]. In addition, owing to the different origin and distinct regulation of thermogenesis in BAT and beigeing in WAT (as beigeing in WAT requires a change in cell fate either by interconversion or by de novo adipogenesis, whereas BAT does not), we can reasonably speculate that mTORC1 signaling may function differently in these two types of adipocytes. However, the underlying mechanism of mTORC1 action in WAT and BAT is still unclear and requires further investigation in the future.

#### Manipulation of up- or downstream regulatory molecules of mTORC1

As we have said, many up- and downstream regulatory molecules participate in the activation or inhibition of mTORC1 signaling cascades either through direct action, or a negative feedback regulation. Studies have shown that knockdown or overexpression of some of these molecules alter mTORC1 signaling activity, thus affecting thermogenesis.

S6K is a direct substrate and effector of mTORC1. When activated, it is involved in the regulation of protein synthesis, cell growth, and proliferation. S6K plays a negative role in IRS-1/PI3K/Akt/mTOR signaling through the phosphorylational suppression of IRS-1 [54–57]. Mice with whole body S6K deficiency show reduced fat mass but enhanced lipolysis, increased WAT mitochondria numbers, and upregulated oxidative phosphorylation and metabolic-related genes, such as Ucp1, Ucp3, CPT1, and PGC-1α in the overnutrition state, implying that mTORC1/S6K signaling plays a negative role in thermogenesis [96]. To date, no S6K adipose-specific ablation mouse model has been used to address its function, a matter which requires further investigation in the future.

4EBPs (a family of the 4EBP protein) are negative downstream effectors of mTORC1. Mice with whole-body double knockout of 4EBP-1 and 4EBP-2 show activated mTORC1 accompanied by accelerated adipogenesis and increased adiposity, reduced oxygen consumption, lipolysis, and energy expenditure under both normal diet and high-fat diet [97]. Similarly, no 4EBPs adipose-specific ablation mouse model has been reported.

Grb10 is the direct substrate of mTORC1, interacts with both insulin receptor and Raptor, and is capable of inhibiting the insulin and mTORC1 signaling pathway via a negative feedback mechanism [55, 56]. Impaired Grb10 expression in mouse adipose tissue (Grb10^Adipoq-Cre^) attenuates core body temperature and cold-induced thermogenic gene expression with increased S6K phosphorylation [57]. In line with this, we have recently discovered that, by using the adiponectin-Cre mouse model, adipose-specific knockdown of Rheb, a direct activator of mTORC1, increases lipolysis and promotes beigeing and energy expenditure by activating the cAMP-PKA-CREB pathway, which results in increased Ucp1 expression in the subcutaneous WAT [98]. These studies further imply that adipose mTORC1 may play a negative role in browning of WAT. However, we have also discovered that Rheb^Adipoq-Cre^ decreases PKA activity and thermogenic gene expression in BAT and Rheb promotes brown fat thermogenesis through the Notch-dependent activation of the PKA signaling pathway [99]. These studies further suggest that thermogenesis in brown and beige fat might be regulated by distinct signaling pathways.

Interestingly, the alteration of mTORC1 activity by another upstream regulator seems to cause opposite results. TSC1 is the negative upstream molecule of mTORC1. Deletion of TSC1 using adiponectin-Cre to constitutively activate mTORC1 enhances mitochondrial activity and fatty acid oxidation, but induces browning and reduces visceral adiposity in mice. However, it has no effect on BAT [100]. Nonetheless, another study found that the activation of mTORC1 signaling by deletion of TSC1 using aP2-Cre leads to significantly increased accumulation of lipid droplets in BAT, downregulated brown adipocyte markers, and upregulated white adipocyte markers [101]. Again, the different results of TSC1 ablation on adipose tissues might be caused by different Cre mice models. As we have mentioned before, aP2-Cre would cause nonspecific tissue deletion and influences from other TSC1-deleted tissues might exist.

Collectively, although the results of adipose TSC1^Adipoq-Cre^ ablation show that overactivation of mTORC1 leads to the browning of WAT [100], the whole-body knockout of S6K [96] or 4EBPs [97] and adipose specific knockout of Grb10 [57] or Rheb [98] seem to imply that mTORC1-related signaling negatively regulates energy expenditure and thermogenesis. Although the use of different experimental systems might result in the discrepancy in thermogenesis caused by deletion of the upstream regulator or the downstream effector of mTORC1, the complexity of the mTORC1 signaling networks may also contribute to the discrepant results, as these molecules can regulate many other signaling pathways and be regulated by them, as well. As possible causes, mTORC1 signaling-dependent and independent mechanisms should not be ruled out and deserve further investigation.

### mTORC2 signaling in brown and beige adipocytes

Unlike mTORC1, little is known about the mTORC2 signaling pathway in brown and beige adipocyte development and thermogenesis.

It seems rational that, in order to compensate for the loss of mitochondrial ATP production due to Ucp1 uncoupling, glucose uptake and glycolysis are stimulated as the energy source during heat production [102]. Current evidence suggests that the beneficial effect of mTORC2 on thermogenesis is in line with its ability to improve glucose uptake. In brown adipocytes, βAR stimulation and cold exposure activate mTORC2 signaling, which, in turn, stimulates cold-induced glucose uptake and glycolysis in vitro and in vivo [103].

To understand the role of mTORC2 in adipocytes, several animal models targeting Rictor, a key component of mTORC2, have been reported. Studies have shown that, using aP2-driven Cre mouse model, Rictor might facilitate glucose uptake that is advantageous to thermogenesis [104–106]. However, as we have discussed before, due to the nonspecific deletion of aP2-Cre, the effects of Rictor ablation on adipocytes and glucose uptake and glycolysis should be further confirmed by specific adipose mouse models.

Studies have shown that, using conditionally deleting Rictor in the Myf5 lineage, Rictor is dispensable for myogenesis and viability, but essential for normal BAT growth [107]. Suppression of the mTORC2 pathway in brown preadipocytes shifts BAT metabolism to a more oxidative and less lipogenic state. Rictor^Myf5-Cre^ ablation in BAT causes higher mitochondrial activity and protects mice from high-fat diet-induced obesity and hepatic steatosis at thermoneutrality, while in acute cold challenge, RictorMyf5-Cre mice show significantly induced Ucp1 expression in BAT [107]. This observation suggests that Rictor/mTORC2 might act as a signaling node that balances oxidative and lipogenic metabolic states.

Studies have shown that, using the adiponectin-Cre mouse model, Rictor^Adipoq-Cre^ deletion in mature adipocytes decreases ChREBPβ expression, reduces de novo lipogenesis (DNL), and impairs hepatic insulin sensitivity, in part by reduced control of glucose uptake through ChREBPβ, suggesting that mTORC2 may regulate ChREBP-driven DNL and hepatic glucose metabolism [108]. However, the effects of Rictor ablation on adipose thermogenesis were not evaluated in the study.

AKT is one of the key downstream effectors of mTORC2. By crossing Akt2 floxed mice with Ucp1-Cre mice to delete Akt2 specifically in BAT, Guertin and colleagues found that Akt2 drives DNL in adipocytes by stimulating ChREBPβ transcriptional activity, and cold induces the Akt2-ChREBP pathway to promote lipid synthesis and oxidation for optimized fuel storage and thermogenesis [109]. In addition, BAT ablation of Akt1 and Akt2 by Myf5- or Ucp1-Cre or Ucp1-CreER mice demonstrates that AKT signaling is required for BAT development and maintenance in vivo [110]. These studies imply that mTORC2-associated signaling plays a positive role via the Rictor-AKT-ChREBP axis in the control of lipid metabolism, thermogenesis and energy expenditure.

Current evidence suggests that the role of mTORC2 signaling in thermogenesis in adipose tissue seems to be correlated with glucose metabolism and lipid oxidation. Apart from the distinct Cre models used in those research studies, the detailed signaling pathways involved in mTORC2 directly or indirectly may also impact thermogenesis. However, as mTORC2 functions with various molecules, it is difficult to determine which aspects have the predominant effect on the comprehensive outcomes, making it arduous to conclude the overall role of mTORC2 in heat production. In order to identify whether mTORC2 is a positive or negative regulator in the thermogenic process, more direct models or methods need to be explored.

## Concluding remarks and future direction

**Fig. 3:**
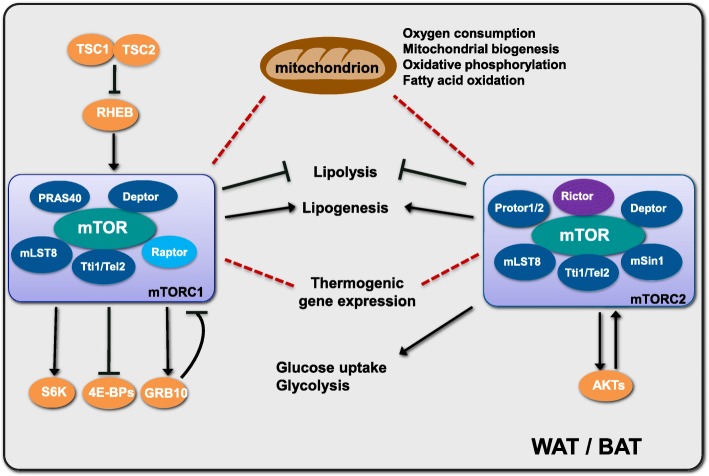
Role of mTORC1 and mTORC2 related signaling in adipose tissues and thermogenesis. The mTORC1 and mTORC2 related signaling pathways play multiple important roles in brown and beige adipocytes and thermogenesis. Current studies show that mTORC1 and mTORC2 related signaling involve in thermogenesis by regulating lipid metabolism (lipolysis and lipogenesis), thermogenic gene expression, and mitochondrial biogenesis and function. Adipose mTORC2 also controls glucose homeostasis by promoting glucose uptake and glycolysis. The triangular arrows suggest positive regulation and the blunt arrows suggest negative regulation. The red dashed lines indicate that both positive and negative roles are reported in different animal models and experimental systems.WAT: white adipose tissue; BAT: brown adipose tissue

mTORC1 and mTORC2 function in diverse signaling pathways to affect heat production separately, and they play complex but crucial roles in the regulation of adipogenesis, lipid metabolism and thermogenesis in adipose tissues [38, 111] (Fig. 3). Given recent studies, paradoxical views of the signaling in adipocytes mostly arise from different mouse models and distinct stimulation conditions. A summary of the manipulation of mTOR-related genes in rodent adipose tissues is provided in Table 1.

**Table 1:** Summary of adipose mTOR signaling on thermogenetic effects in rodents

Refs	mTOR related molecules	Mouse model/ exp. condition	Activity of mTOR signaling	Tissue affected	Phenotypes
Liu et al.2016 [79]		Rapamycin-treated mice (7days) under cold exposure Rapamycin-treated mice (2 days) under cold exposure	mTORC1 inhibited	WAT BAT	Thermogenic gene expression ↓ Thermogenic gene expression ↓ Core body temperature ↓
Tran et al.2016 [80]		Rapamycin-treated mice (2 weeks) under CL Stimulation (24 hours)	mTORC1 inhibited	WAT	Cold tolerance ↓ Thermogenic gene expression ↓ Beige fat gene expression ↓
Makki et al.2014 [81]		Diet-induced obese mice with chronic treatment of rapamycin (22 weeks)	mTORC1 inhibited	BAT	Energy expenditure ↑ Oxygen consumption ↑ BAT activity ↑
Labbe et al.2016 [71]	Raptor	raptor-/- mice (Adipoq-Cre) under cold exposure (2 weeks)	mTORC1 inhibited	BAT	Systemic oxygen consumption ↓ BAT mass ↓ Nucleotide synthesis ↓ Mitochondrial biogenesis ↓ TCA cycle activity ↓ Thermogenic gene ↓
Lee et al. 201 6[72]	Raptor	raptor-/- mice (Adipoq-Cre) with HFD	mTORC1 inhibited	WAT BAT	Expression of Ucp1 in WAT ↑ Expansion of WAT and BAT ↓ No changes on energy expenditure
Liu et al.2016 [79]	Raptor	raptor-/- mice (Adiponq-Cre) under cold exposure (7 days)	mTORC1 inhibited	WAT BAT	Expression of Ucp1 ↓ Mitochondrial related genes ↓
Tran et al.2016 [80]	Raptor	raptor-/- mice (Adiponq-Cre) with CL stimulation (24h hours)	mTORC1 inhibited	WAT	Thermogenic gene ↓ Lipolysis ↓ Adipocyte size ↑
Polak et al.2008 [86]	Raptor	raptor-/- mice (aP2-Cre) at thermoneutrality	mTORC1 inhibited	WAT	Oxygen consumption ↑ Ucp1 expression ↑
Zhang et al.2018 [87]	Raptor	raptor-/- mice (Adiponectin-Cre) under cold exposure (2 days)	mTORC1 inhibited	WAT BAT	Beige adipogenesis ↑ Thermogenic gene ↑ Thermogenic gene ↓
Shan et al.2016 [73]	mTOR	mTOR-/- mice (Adipoq-Cre) with ND	mTORC1 inhibited	WAT BAT	Mass of BAT and WAT ↓ Thermogenic genes in WAT ↑ No changes on energy expenditure
Um et al.2004 [96]	S6K	S6K1-/- mice (whole body) under ND or HFD	mTORC1 inhibited	Whole body	Lipolysis in WAT ↑ Mitochondria numbers in WAT ↑ Oxidative phosphorylation in WAT ↑ Metabolic related genes in WAT ↑
Le Bacquer et al. 2007 [97]	4EBPs	4E-BP1/ 2-/- mice (whole body) under ND or HFD	mTORC1hyper-activated	Whole body	Oxygen consumption ↓ Lipolysis in WAT ↓
Liu et al.2014 [57]	Grb10	Grb10^-/-^mice (Adipoq-Cre) under cold exposure	mTORC1hyper-activated	BAT	Core body temperature ↓ Cold-induced thermogenic genes ↓
Meng et al.2017 [98] [99]	Rheb	Rheb^-/-^mice (Adipoq-Cre) under ND or HFD, with or without cold exposure	mTORC1 inhibited	WAT BAT	Lipolysis ↑ Beige adipocytes in WAT ↑ Energy expenditure ↑ Thermogenic gene expression in BAT ↓
Magdalon et al.2016 [100]	TSC1	TSC1-/- mice (Adipoq-Cre) under ND	mTORC1hyper-activated	WAT	Lipolysis ↑ Ucp1 expression ↑ Mitochondrial oxidative activity ↑ Fatty acid oxidation ↑ PGC-1α and PPARα
Xiang et al.2015 [101]	TSC1	TSC1-/- mice (aP2-Cre) under ND	mTORC1hyper-activated	BAT	Brown adipocyte genes ↓ White adipocyte genes ↑
Albert et al.2016 [103]	Rictor	rictor-/- mice (aP2-Cre) under cold exposure	mTORC2 inhibited	BAT	Cold tolerance ↓ Glucose uptake ↓ Glycolysis ↓
Kumar et al.2010 [104]	Rictor	rictor-/- mice (Ap2-Cre)	mTORC2 inhibited	BAT WAT	Glucose uptake ↓ Glycolysis ↓
Hung et al.2014 [107]	Rictor	rictor-/- mice (Myf5-Cre) under cold exposure	mTORC2 inhibited	BAT	Mitochondrial activity ↑ Ucp1 expression ↑
Tang et al.2016 [108]	Rictor	rictor -/- mice (Adipoq-Cre)	mTORC2 inhibited	WAT	Lipogenesis ↓ Glucose uptake ↓
Sanchez et al.2018 [109]	Akt2	Akt2-/- mice (Ucp1-Cre) under cold exposure	mTORC2 inhibited	BAT	Lipid synthesis and oxidation ↓ Ucp1 expression ↓
Sanchez et al.2019 [110]	Akt1 Akt2	Akt1and Akt2-/- mice (Ucp1-Cre/Ucp1-CreER/Myf5-Cre)	mTORC2 inhibited	BAT	Lipid droplets in BAT ↓ Ucp1 expression ↓

Abbreviations: *BAT* Brown adipose tissue, *CL* CL-316243, *Grb10* Growth factor receptor-bound protein 10, *HFD* High fat diet, *ND* Normal diet, *Raptor* Regulatory associated protein of mTOR, *Rheb* Ras homolog enriched in brain, *Rictor* Raptor-independent companion of mTOR, *S6K* Ribosomal S6 kinase, *TSC1* Tuberous sclerosis complex 1, *WAT* White adipose tissue, *4E-BPs* Eif4e-binding proteins

Note: ↑ increased; ↓ decreased

The proportion of classic and nonclassical pathways of mTOR-related signaling involved in thermogenesis force us to focus on the primary section or molecules, providing a more valid and valuable treatment. Because the activation of mTOR in adipose tissues under distinct stimulations may be involved in diverse molecules, it is reasonable to expect that the role of mTOR in thermogenesis depends on distinct circumstances and the energy status of cells. Further investigation is needed, which requires us to use more appropriate and valuable animal models and/or identical experimental conditions, including environmental temperature and nutrient utilization, to clarify the effects of the mTOR signaling pathways on thermogenesis. In addition, the cross-talks between mTORC1 and mTORC2 in adipocyte metabolism and thermogenesis would also be interesting to explore.

Because of the distinct origin and features of brown and beige adipocytes, more investigation is needed to compare the consequences and underlying mechanisms of the two types of adipocytes in response to mTOR manipulation. As mTOR signaling regulates development and its embryonic ablation might have an impact on the development of animals, temporal control of adipose-specific recombination is important. Using a more specific Cre mouse model, such as the tamoxifen-inducible CreERT2 line, is required for future studies. In addition, with the development of BAT (e.g., Ucp1-Cre lines [112]) or beige-specific Cre lines, or with a newly developed Ucp1-CreER line [31], one should allow for specific and temporal control of recombination in either brown or beige fat, respectively.

Altogether, despite a few contrary viewpoints on mTOR signaling in thermogenesis in adipose tissues, and although it is hard to determine the role of mTOR, considerable progress has revealed novel insights into the mechanisms and functions of mTOR in heat production. Further comprehensive understanding of the role and underlying mechanisms of mTOR signaling in thermogenesis in adipose tissues under certain energy status and environmental conditions will be of great significance for future therapeutic and medical interventions for obesity and related metabolic disorders.

## Data Availability

Not applicable

## Abbreviations

4E-BP: eIF4E-binding protein
AGRP: Agouti-related neuropeptide
AKT: Protein kinase B
AMPK: AMP-activated protein kinase
ATF4: Activating transcription factor 4
ATP: Adenosine triphosphate
BAT: Brown adipose tissues
C/EBPβ: CCAAT/enhancer-binding protein-β
cAMP: Cyclic adenosine monophosphate
CIDEA: Cell death inducing DFFA like effector A
CITED1: cbp/p300-interacting transactivator with Glu/Asp rich carboxy-terminal domain 1
CPT1: Carnitine palmitoyltransferase 1
DEPTOR: DEP domain-containing mTOR-interacting protein
DIO2: iodothyronine deiodinase 2
EBF2: Early B-cell factor 2
EHMT1: Euchromatic histone-lysine N-methyltransferase 1
EN1: Engrailed 1
EPAC1: Exchange protein directly activated by cAMP 1
FKBP12: 12-kDa FK506- and rapamycin-binding protein
FLCN: Folliculin
FRB: FKBP-rapamycin binding
GLUT1: Glucose transporter 1
GLUT4: Glucose transporter 4
GRB10: Growth factor receptor–bound protein-10
GSK3β: Glycogen synthase kinase 3β
HIF1α: Hypoxia inducible factor-1α
HOXC8: Homeobox C8
HOXC9: Homeobox C9
HSL: Hormone-sensitive lipase
IGFBP2: Insulin-like growth factor–binding protein 2
IMP1: IGF2 mRNA-binding protein 1
LATS: Large tumor suppressor homologue kinase
mLST8: mammalian lethal with SEC13 protein 8
mSIN1: mammalian stress-activated protein kinase-interacting protein 1
MST1: mammalian sterile 20-like kinase 1
mTOR: mechanistic target of rapamycin
MYF5: Myogenic factor 5
MYH11: Myosin heavy chain 11
NP: Natriuretic peptide
NPY: Neuropeptide Y
PAX7: Paired box 7
PDE3B: Phosphodiesterase 3B
PDGFRα: Platelet-derived growth factor receptor-α
PDGFRβ: Platelet-derived growth factor receptor-β
PGC-1α: PPARγ- coactivators-1α
PGC-1β: PPAR-γ coactivator 1-β
PGs: Prostaglandins
PI3K: Phosphoinositide 3-kinase
PKA: Protein kinase A
PKC: Protein kinase C
PPARα: Peroxisome proliferators-activated receptors α
PPARγ: Peroxisome proliferator-activated receptor γ
PRAS40: Akt/PKB substrate 40 kDa
PRDM16: PRD1-BF1-RIZ1 homologous domain-containing 16
PROTOR1/2: Protein observed with rictor-1 and -2
PTEN: Phosphatase and tensin homolog
RAG: RAS-related GTP-binding protein
RAPTOR: Regulatory associated protein of mTOR
RHEB: Ras homolog enriched in brain
RICTOR: Raptor-independent companion of mTOR
RYR2: Ryanodine receptor 2
S6K: S6 kinase
SERCA2b: Sarco endoplasmic reticulum calcium ATPase 2b
SGK1: Serum- and glucocorticoid-induced protein kinase 1
SMA: Smooth muscle actin
STAT6: Signal transducers and activators of transcription 6
TAG: Triacylglycerol
TCA: Tricarboxylic acid cycle
TFE3: Transcription factor binding to IGHM enhancer 3
TFEB1: Transcription factor EB1
Tregs: Regulatory T-cells
TSC 1/2: Tuberous sclerosis complex 1/2
UCP1: Uncoupling protein 1
UCP3: Uncoupling protein 3
ULK1: UNC-51-like kinase 1
WAT: White adipose tissues
YAP: Yes-associated protein
ZFP516: Zinc finger protein 516
βAR: β-adrenergic receptor
